# The role of brain radiotherapy for EGFR- and ALK-positive non-small-cell lung cancer with brain metastases: a review

**DOI:** 10.1007/s11547-023-01602-z

**Published:** 2023-02-14

**Authors:** Valerio Nardone, Caterina Romeo, Emma D’Ippolito, Pierpaolo Pastina, Maria D’Apolito, Luigi Pirtoli, Michele Caraglia, Luciano Mutti, Giovanna Bianco, Antonella Consuelo Falzea, Rocco Giannicola, Antonio Giordano, Pierosandro Tagliaferri, Claudia Vinciguerra, Isacco Desideri, Mauro Loi, Alfonso Reginelli, Salvatore Cappabianca, Pierfrancesco Tassone, Pierpaolo Correale

**Affiliations:** 1 Department of Precision Medicine, University of Campania “L. Vanvitelli”, 80138 Naples, Italy; 2 Medical Oncology Unit, “Bianchi Melacrino Morelli” Grand Metropolitan Hospital, Reggio Calabria, Italy; 3 Radiotherapy Unit, University Hospital of Siena, Siena, Italy; 4 Sbarro Institute for Cancer Research and Molecular Medicine and Center of Biotechnology, College of Science and Technology, Temple University, Philadelphia, PA 19122 USA; 5 Department of Medical Biotechnologies, University of Siena, 53100 Siena, Italy; 6 Department of Experimental and Clinical Medicine, Magna Græcia University, 88100 Catanzaro, Italy; 7 Neurology Unit, University Hospital of Salerno, Salerno, Italy; 8 Radiation Oncology, Azienda Ospedaliero–Universitaria Careggi, University of Florence, Florence, Italy

**Keywords:** Non-small cell lung cancer (NSCLC), Brain metastases (BM), Central nervous system (CNS), EGFR driver mutation, ALK rearrangement, Tyrosine kinase inhibitors (TKI), ALK inhibitors, Radiotherapy

## Abstract

Non-small cell lung cancer (NSCLC) is frequently complicated by central nervous system (CNS) metastases affecting patients’ life expectancy and quality. At the present clinical trials including neurosurgery, radiotherapy (RT) and systemic treatments alone or in combination have provided controversial results. CNS involvement is even more frequent in NSCLC patients with EGFR activating mutations or ALK rearrangement suggesting a role of target therapy in the upfront treatment in place of loco-regionals treatments (i.e. RT and/or surgery). So far clinical research has not explored the potential role of accurate brain imaging (i.e. MRI instead of the routine total-body contrast CT and/or PET/CT staging) to identify patients that could benefit of local therapies. Moreover, for patients who require concomitant RT there are no clear guidelines on the timing of intervention with respect to innovative precision medicine approaches with Tyrosine Kinase Inhibitors, ALK-inhibitors and/or immuno-oncological therapies. On this basis the present review describes the therapeutic strategies integrating medical and radiation oncology in patients with metastatic NSCLC (mNSCLC) adenocarcinoma with CNS involvement and EGFR activating mutations or ALK rearrangement.

## Background

Non-small cell lung cancer (NSCLC) is the first and second most frequent cause of death from cancer in men and women, respectively.

Adenocarcinoma is the most represented histology with increasing incidence in western countries (> 50%) [1]. Patients diagnosed in advanced or metastatic stage (mNSCLC) have poor prognosis with less than 5% of them surviving more than 5 years [2, 3]. The increased incidence of brain metastases (BMs) is likely resulting from longer patient survival due to more effective systemic therapies for the primary cancer and increased use of neuroimaging in neurologically asymptomatic patients that has allowed prompter treatments of this subset of patients [4, 5].

Before molecular targeted therapy and immune-checkpoint inhibitors monoclonal Antibodies (ICI moAbs), standard treatment was chemotherapy doublet with platinum (either cisplatin or carboplatin) and a second chemotherapeutic drug arbitrarily chosen among gemcitabine, paclitaxel, vinorelbine or pemetrexed eventually combined with anti VEGF mAbs (bevacizumab) (the latter two options restricted to non-squamous histology) [6–9].

Thanks to the detection of EGFR gene alterations and ALK-rearrangements (10–30 and 3–7% of patients, respectively) and other driver mutations critical for lung cancer tumorigenesis and promotion, we have entered a new era of personalized therapy in the treatment of lung cancer patients driven by genotyping [10–12].

Despite these breakthroughs in the treatment of advanced mNSCLC, several points still remain open, in particular for patients who present “ab initio” or develop late BMs [13]. It is noteworthy the BMs are detected in 24.4% in EGFR-mutation patients and 23.8% in ALK-rearrangements patients at the time of diagnosis and respectively 46.7% and 58.4% within 3 years from the diagnosis [14].

Therefore, the present review aims to describe the multidisciplinary strategies in patients with mNSCLC adenocarcinoma with CNS involvement and EGFR activating mutations or ALK rearrangement.

## The medical oncologist point of view

### Frequency of BMs in EGFR/ALK mutant NSCLC

The detection of synchronous BM during the staging of NSCLC is a challenging event for the clinical management of these patients. A recent epidemiological study conducted by Surresh K. et al. suggests a greater incidence of synchronous BM in NSCLC patients bearing *EGFR*/*ALK driver mutation/translocations* compared to other patients’ subsets (62% vs 57%, respectively; *P* < 0.05) with median survival not exceeding 14.6 months. EGFR-activating mutation mainly occurs in younger women [15] and never-smokers [16, 17] with adenocarcinoma histology. These patients have a 50–70% high risk of BMs and about one third of them develops CNS progression during the course of treatment [18]. Additionally, the risk of CNS relapse appears to be higher in patients bearing the L858R point mutations [19]. Interestingly, it seems that the type of EGFR mutation is more related to specific patterns of BMs as suggested by a recent retrospective radiologic analysis of 57 NSCLC patients that recorded a multi-nodular BM pattern in patients bearing an exon 19 deletion [20].

On the other hand, ALK rearrangement is rare and it is detected approximately in 3–7% of patients with the diagnosis of NSCLC [21]. Likewise to EGFR mutations, ALK rearrangement is recorded in young, non-smoking men with non-squamous histology, who are susceptible of treatment with crizotinib, an ATP-competitive, orally bioavailable ALK inhibitor, firstly employed for the treatment of eml4-alk positive NSCLC [22].

Unfortunately, nearly one third of the patients bearing an ALK rearrangement and receiving crizotinib develop CNS metastases within one year of therapy sometimes as the only extra-thoracic site of tumor progression. In this context, the development of second- and third-generation ALK inhibitors such as alectinib in the front line and lorlatinib in treatment lines following the first has encountered greater effectiveness in terms of intracranial response and better outcomes for these patients, overcoming the mechanisms of resistance to crizotinib [23, 24].

## Role of TKI in mNSCLC patients with EGFR mutations and BMs

The management of BM with systemic anticancer drugs presents great limitations due to the presence of a functional Blood–Brain Barrier (BBB) while loco-regional interventions (surgery and radiation therapy) can also damage the adjacent healthy tissue.

Treatments with the first- and second-generation EGFRTKI, including erlotinib/gefitinib and afatinib top the response rate, PFS and survival obtained with doublet chemotherapy. More recently, osimertinib has emerged as an active third-generation EGFR TKI in the front-line setting as well as in patients with T790M mutation responsible for acquired resistance to the other EGFR-TKIs or with CNS lesions [25, 26].

Selected studies reported very promising activity of EGFR-TKIs use in fit patients with BMs, (intracranial response rate of 75% -88%, and median intracranial PFS and OS of 6.6–14.5 and 15.9–21.8 months, respectively) [27–29].

The progressive better understanding of EGFR mutations in mNSCLC has allowed to set up the Lung Cancer Molecular Markers Graded Prognostic Assessment (Lung-mol-GPA based on EGFR status as the main target combined with other clinical parameters (25) to help clinical decisions on newly diagnosed with BM (see Table 1).

**Table 1:** – Lung-molGPA (Lung Cancer Molecular Graded Prognostic Assessment)

Prognostic factor	Age (years)	KPS	Extracranial metastases	Number of BM	Gene status
0	≥ 70	< 70	Present	> 4	EGFR Neg/ukn and ALK neg/ukn
0.05	< 70	70–80	–	1–4	NA
1	–	90–100	Absent	NA	EGFR-pos or ALK-pos

*KPS* Karnofsky performance status, *NA* not applicable, *neg/unk* negative or unknown, *pos* positive, *BM* brain metastases, *EGFR* epidermal growth factor receptor, *ALK* anaplastic lymphoma kinase

It is noteworthy that the efficacy of EGFR TKIs in patients with BM is not clearly as curtained in patients with symptomatic or uncontrolled BM because this patients’ subset was mostly excluded from pivotal, randomized controlled trials. The data concerning a potential efficacy of EGFR-TKI therapy in patients with mNSCLC, mutated EGFR and BMs have been mostly assumed from retrospective studies or indirect evidence.

First-generation TKIs (gefitinib, erlotinib) reversibly blocks EGFR receptor and achieves a mean survival time of 33.1 months. This implies more likely onset of CNS disease, cutting life expectancy to 5.1 months from the diagnosis of BM. Despite their low molecular weight, the incomplete penetration through the BBB is responsible of the low CNS concentration and worse prognosis of gefitinib and erlotinib in these patients [19]. Afatinib is a second-generation TKI that irreversibly binds to the EGFR receptor with higher affinity compared to first-generation TKIs. Two studies Lux-Lung 3 and Lux-Lung 6 [30, 31] have demonstrated the superiority of afatinib over platinum-based doublets also in patients with asymptomatic BMs. The Lux-Lung 7 trial compared gefitinib to afatinib including patients with BMs [32]. Despite its promise as second-generation irreversible EGFR targeted agent, afatinib showed no superiority over the first-generation agents (except in some of the less common EGFR mutations) and less manageable toxicity profile.

Osimertinib is a further EGFR TKI resulted very active in mNSCLC/EGFR mut patients who developed the EGFR T790M mutation [33] known to be the most common mechanism of resistance to first- and second-generation TKI in 50–60% of patients who show progression [17]. Osimertinib efficacy also showed superiority over chemotherapy in this subset of patients with BMs [34–36]. The efficacy of osimertinib in EGFRmut mNSCLC was demonstrated by the results of the AURA 3 trial [37] and subsequently confirmed in the FLAURA trial where it resulted also superior to first-generation EGFRTKI in term of PFS and OS [35, 36, 38, 39].

In particular the mean response time in the CNS reported in the AURA 3 trial was 8.9 months with Osimertinib versus 5.7 months with chemotherapy [34, 40]. Moreover, FLAURA clinical trial similarly showed the efficacy of osimertinib in patients with CNS metastases [36, 41]. Interestingly, within this trial it was shown that the presence of the uncommon C797SEGFR mutation was strongly predictive of resistance to osimertinib [41, 42] opening for the research of further drugs able to overcome this mechanism of resistance. Nevertheless, the antitumor effects of osimertinib single agent on CNS metastasis are unclear because these studies included patients treated with RT whose effects can be tardive.

The OCEAN study was a two-cohort trial showing the efficacy of osimertinib in achieving BM response rate (BMRR) in RT-naïve patients with T790M EGFR mutated NSCLC especially in the presence of exon 19 deletion [43]. Another interesting drug in this setting is represented by AZD3759, a miscellaneous oral EGFR TKI designed for CNS penetration that caused tumor regression in leptomeningeal and BM mouse models [44]. Preliminary results of the phase I BLOOM study of 38 EGFR-mutant NSCLC with BM or leptomeningeal metastasis (LM) treated with AZD3759 showed an intracranial ORR of 63% [44]. Table 2 summarizes the prospective trials of three generations of EGFR TKI in EGFR-mutant NSCLC with BM.

**Table 2:** Prospective studies in EGFR mutant NSCLC patients with BM

Study	TKI	EGFR mutant NSCLC patients with BM	RR (%)	Survival (months)	CNS ORR (%) Duration of CNS control (months)
Park 2012, phase II	Erlotinib or Gefitinib	28	PR = 83; SD = 11	PFS = 6,6; OS = 15,9	Not assessed
Yu 2017, phase I	Pulsatile Erlotinib	34 (only 32% had BM)	CR = 2, PR = 70	PFS = 9,9	No patient had progression of an untreated CNS metastasis or developed a new CNS lesion while on study (0%, 95% CI 0–13%)
luchi 2013, Phase II	Gefitinib	41	ORR = 87.8	PFS = 14,5; OS = 12,9	Response of BM (%) CR 31.7% PR 56.1% CR + PR 87.8% SD 9.8% PD (2.4%) The CNS RR of tumors with exon 19 deletion was superior to those with L858R (100% vs 80%) 14.5 mo
Yang 2017 (BRAIN), Phase III)	Icotinib	85	–	Intracranial PFS = 10	HR for intracranial disease progression or death 0·56, 95% CI 0·36–0·90; *p* = 0·014)
Schuler 2016 (LUX-Lung 3/6), Phase III	Afatinib	35/46	–	PFS = 11,1–8,2	CNS ORR 23 of 28 (82.1%) and 12 of 20 (60.0%) in those with Del19 or L858R mutations, respectively Additionally, in patients with uncommon EGFR mutations and brain metastases, ORR was observed in 3 of 9 patients (33.3%)
Park 2016 (Lux-Lung 7), Phase II	Afatinib	26	–	ORR 70 CR 1 PR 69 SD 21 PD 6 DC 91 8,4	Not assessed
Mok 2017 (AURA 3), Phase II	Osimertinib	144 (T790M)	–	PFS = 10,1	Mean response time for CNS metastases: 8.9 months
Goss 2017 (AURA, AURA2), Phase II	Osimertinib	50 (T790M)	CNS ORR = 54	–	54 Median CNS duration of response (22% maturity) was not reached (range, 1–15 months); at 9 months, 75% (95% CI 53–88) of patients were estimated to remain in response. Median follow-up for CNS PFS was 11 months; median CNS PFS was not reached (95% CI, 7, not calculable)
Yang 2017 (BLOOM), Phase I	Osimertinib	32 (LM, 11 T790M)	ORR = 43	–	LM ORR 63% LM DoR 15.2 mo
Soria 2018(FLAURA), Phase III	Osimertinib	53	ORR = 75; CNS PD = 6	CNS PFS = 15,2	Not assessed
Yamaguchi 2021 (OCEAN), Phase II	Osimertinib	66	ORR = 40.5% BMRR = 70%	PFS = 25.2 OS = 19.8	BMRR 66.7 median BMs PFS 25.2 mo

*PR* partial response, *SD* stable disease, *CR* complete response, *PFS* progression free survival, *OS* overall survival, *ORR* objective response rate, *CNS* central nervous system, *BMRR* brain metastases response rate, *LM* leptomeningeal

## Role of the newest molecular target therapies in mNSCLC patients with ALK rearrangements and BM

The EML4/ALK fusion gene is a rare mutation occurring in 3–7% of mNSCLC that induces the constitutive activation of the ALK tyrosine kinase and downstream pathways [45]. This subset of patients with CNS involvement results highly responsive to the frontline treatment with ALK-TKI.

Crizotinib was the first ALK-TKI approved in these patients based on the successful results of the phase 3 Profile 1014 study [22, 46]. Not with standing CNS relapse resulted approximatively 30% more frequent with crizotinib than with chemotherapy within the first year of treatment [47].

In the ALEX phase 3 clinical trial alectinib, a second-generation ALK-TKI was compared to crizotinib in first-line treatment of metastatic ALK-positive NSCLC showing a longer PFS and brain control [24]. During the first 12 months incidence of CNS progression with alectinib or crizotinib treatment was, respectively, 9.4% versus 41.4%. Alectinib showed a better intracerebral disease control with an average PFS of 25.7 months than that of 10.4 months recorded for crizotinib [24]. Further studies detected multiple resistance mutations responsible for the treatment failure with ALK-TKI including the I117N which confers tumor resistance to alectinib. This resistance, however, may be overcome by the use of ceritinib [48, 49]. When evaluated in the phase 3 clinical trial ASCEND-4 vs doublet chemotherapy as a frontline therapy in patients with BMs bearing ALK rearrangement, ceritinib achieved a better reduction of measurable CNS lesions (72.7% vs. 27.3%) [50]. Additionally, the ASCEND-1 trial in patients with ALK rearrangement recorded a total intracerebral ORR of 63% in naïve patients and 36% in mNSCLC who had received ceritinib as a salvage therapy after previous treatment lines with other ALK TKIs [51]. These results were mostly confirmed in the ASCEND-2 trial where the use of ceritinib resulted in an intracerebral ORR of 85% in chemo-naive patients and 40% in those who had received previous ALK-TKI lines [52]. AG1202R is another well-known ALK mutation, conferring resistance to either first- or second-generation ALK-TKIs and potentially overcome using the newest TKIs brigatinib and lorlatinib. Both drugs have in fact been designed for their ability to penetrate the BBB and to overcome the resistance to TKIs approved for frontline treatment. Naito T. and colleagues have recently reviewed the substantial activity of brigatinib in controlling CNS metastases, in crizotinib-treated (ALTA trial) patients and crizotinib-naïve (ALTA-1L trial) patients with ALK rearrangement with or without specific resistance mutations. They also reported an analogue activity of lorlatinib in NSCLC patients with intracranial lesions bearing ALK, or c-ros oncogene 1 (ROS1)-positive rearrangements/mutations [53].

Thanks to its activity against ALK-G1202R mutation (responsible for resistance to first- and second- generation ALK inhibitors) lorlatinib is a valid therapeutic option. Updated results from the Phase 3 CROWN trial, which evaluated lorlatinib versus crizotinib in people with previously untreated (ALK)-positive advanced NSCLC, reported that after a median follow-up of three years lorlatinib continues to demonstrate meaningful improvement in PFS compared to crizotinib (HR, 0.27; 95% CI, 0.18–0.39), corresponding to a 73% reduction in the rate of progression or death. Moreover, lorlatinib treatment resulted in a 92% reduction in the rate of intracranial progression (HR, 0.08; 95% CI, 0.04–0.17). The intracranial objective response rate (IC-ORR) for people with measurable BM at baseline was 83% (95% CI, 59–96, *n* = 15) with lorlatinib and 23% (95% CI, 5–54, *n* = 3) with crizotinib, with an intracranial complete response rate of 72% and 8%, respectively. In people without BMs at baseline, lorlatinib demonstrated a 98% reduction in the rate of intracranial progression (HR 0.02; 95% CI, 0.002–0.136). Finally, the long-term results from the CROWN trial confirm lorlatinib compelling safety and efficacy profile in the first-line setting and sustained benefit for up to three years for this patient population [3, 14, 54].

Table 3 summarizes the prospective trials of three generations of ALK inhibitors in ALK-rearranged NSCLC with BMs.

**Table 3:** Prospective studies in ALK-rearranged NSCLC patients with BMs

Study	ALK inhibitor	Number of patients with BMs	RR (%)	Survival (months)	CNS ORR (%) Duration of CNS control (months)
PROFILE 1014, Solomon 2014, phase III	Crizotinib	92	ORR 74%	PFS 10.9 mo (HR = 0.45) OS Not reached HR = 0.82	CNS PFS HR 0.57
ASCEND 1, Kim 2016, phase I	Ceritinib	124	ORR 72.3% for ALKI-naive and 56.4% for ALKI-pretreated	PFS 18.4 mo in ALKI-naïve and 6.9 mo in ALKI-pretreated	Median intracranial DOR 6.9 mo CNS ORR 78.9% in ALKI-naïve and 65.3% in ALKI-pre treated
ASCEND 2, Mok 2015, phase II	Ceritinib	100	54%	BM PFS 5.4 mo	IC ORR 85% (naïve) and 40% DOR 9.2 mo
ASCEND 4, Soria 2017, phase III	Ceritinib	121		16.6 mo	CNS PFS 10.7 mo IC ORR 72.7% vs 27.3%
ALTA-1-L, Ross 2020, phase III	Brigatinib	47	ORR 74%	PFS 24 mo	CNS ORR 66% (78% in measurable BMs) DOR 24 mo
J-ALEX, Hida, 2017, phase III	Alectinib	29	ORR 92%	PFS NR	CNS PFS HR 0.16
ALEX, Peters 2017, phase III	Alectinib	58	26%	PFS 25.7 mo	26% CNS cumulative events (progression) 9.4% vs 41.4% 3.6 months
ALESIA, Zhou 2019, phase III	Alectinib	44	ORR 91%	PFS NR	CNS ORR 73% CNS PFS HR 0.14
CROWN, Shaw 2020, phase III (Updated results)	Lorlatinib	38	76%	PFSHR 0.27 HR for intracranial progression 0.07 HR for OS 0.72	IC-ORR in patients with measurable BM 83% (CR 72%); IC-ORR in patients without BM at baseline 98%)

*PFS* progression free survival, *OS* overall survival, *HR* hazard ratio, *IC* intracranial, *ORR* objective response rate, *CR* complete response, *DOR* duration of response, *mo* months, *CNS* central nervous system, *BM* brain metastases, *NR* not reached

## The radiation oncologist point of view

The use of radiation therapy/radiosurgery and/or surgery remains the backbone of BM management in mNSCLC patients due to the low permeability of BBB to most of the conventional anticancer drugs. Nevertheless this statement has been partially challenged for patients with oncogene-driven NSCLC.

Currently whole brain radiotherapy (WBRT) and the focal radiotherapy are integrated with either surgery or systemic therapies within a multimodal approach.

WBRT has been the standard approach to treatment of BMs from NSCLC thanks to an improvement of symptoms and distant BM control, in 70–93% and 60–80% of patients, respectively [55–57].

The neurocognitive toxicity, and the lack of impact on the survival of mNSCLC with BMs has determined a progressive decline of WBRT in favor of less invasive strategies including stereotactic radiosurgery (SRS).

In a phase III study WBRT and SRS equally affected OS, but SRS caused less decline in neurocognitive function (WBRT plus SRS 53% vs. 20% SRS alone), and an increased risk of further intracranial relapse [56]. This risk, however, could be theoretically counterbalanced by a strict follow-up and new salvage SRS on recurrent BMs. Furthermore, appropriate systemic therapy may delay further intracranial progression, as more recently observed in patients with mNSCLC receiving multimodal treatment with SRS and immunotherapy [58].

Therefore, mNSCLC patients with BM should be evaluated within a competent multidisciplinary team. Surgery may be offered for patients with solitary large brain metastases to counteract the expanding mass effect in the CNS whereas (despite the impact of multiple significant co-variables) in patients with a single BM SRS and surgery are equally effective on LR and OS [61].

It is noteworthy that patients with BMs require supportive car to prevent and treat the frequent complications (i.e. cerebral edema, epilepsy, pain, etc.) and this should drive the decision making prior to combining ablative therapy and EGFR-TKI.

A major argument against the use of brain RT encourages the use of the newest anticancer drugs in mNSCLC that, on one hand, overcome the BBB with no damage of healthy CNS (i.e. radio-necrosis) and on the other hand obtain satisfactory intracranial disease control [59]. However, it cannot be ruled out that upfront BM treatment with locoregional treatment could prevent in selected patients on TKI with expected long survival.

### Treatment strategies based on BM numbers and dimension

Brain oligometastatic disease is a common scenario in which the number of brain lesions becomes a “moving target” whose management is still far to be established. Patients with a single metastatic brain lesion experience significantly longer survival with minimal cognitive impairment and CNS symptoms (other than seizures) compared to patients with multiple metastases. Moreover, it has been shown that postoperative radiotherapy may significantly reduce the risk of local recurrence, whereas combined use of the two locoregional treatments improves the neurologic control of disease and the survival of these patients [60, 61]. Although WBRT has been long recognized as the standard adjuvant procedure after BM resection, a large phase III trial revealed a longer cognitive-deterioration-free survival of patients on SRS compared to WBRT with comparable effects of the two treatments in term of OS [62]. A further study compared the effects of SRS focused on the surgical cavity in patients with radical resection of 1–3 BMs and revealed that the prophylactic radiotherapy reduced the local recurrence rate at 12 months with no effects on OS [60]. The results of the two trials prompted the adoption of SRS as the new standard after surgical resection of BMs [63]. The BM scenario is still more complex in mNSCLC patients with specific oncogene addiction. The results of recent studies in mNSCLC in fact suggest a significant heterogenicity in the expression (about 20%) of EGFR mutations with great discordance recorded between primary tumor and brain lesions [64, 65]. Therefore a further brain biopsy to confirm the presence EGFR mutations also in the brain lesions should be recommended to define a personalized treatment strategy including SRS.

A rising number of recent studies focus on the comparison of WBRT vs. SRS and indicate that SRS is an important alternative to WBRT in fit patients. Japanese researchers reported the results of the prospective JLGK0901 trial indicating that SRS is still relevant in the presence of more than three CNS lesions [66]. The use of SRS was associated to a median OS of 13.9 months (455 cases) 10.8 months (531 cases) and 10.8 months (208 cases) in patients with single BM, 2–4 treated BMs, and of 5–10 treated BMs, respectively.

However, a retrospective study conducted by Balasubramanian et al. [67] showed that the use of target therapy along with surgery and/or radiation may improve the OS on EGFR mut mNSCLC patients regardless the number of BMs.

SRS and more conservative strategies are gaining further field of application also in large brain metastases with a diameter of > 2 cm. Patients with large BMs, commonly present severe neurologic invalidating symptoms and/or significant vasogenic edema or mass effect requiring fast upfront surgical resection when feasible. The subsequent post-operative SRS (median dose 15 Gy) after GTR, with average volume of 8.7 e 9.6 mL, can improve both LC and OS [68, 69]. Drawbacks to this treatment are always possible as neurological complication because of extensive resection and risk of symptomatic radionecrosis associated to ample planning target volume margin size (> 1.0 mm) for SRS [70, 71]. Jhaveri et al. carried out a multivariate analysis in mNSCLC, whose results showed that a GTV > 15 cc is the main risk factor predictive of local recurrence [70]. Additionally, volumes of healthy brain tissue larger than > 10 mL receiving 12 Gy (V12 Gy) are directly correlated with radionecrosis (between 15 and 55%) [72–74]; hence the use of fractionated SRS (fSRS: i.e. V12 > 8.5 ml (30 Gy/5 fx; 27 Gy/3 fx) is advised in order to reduce this risk still maintaining an improvement of LC especially when the BM lesions are located in or near eloquent areas [73]. At this purpose, the A071801 phase III trial aimed to evaluate the efficacy of SRS compared with fSRS for resected BMs in mNSCLC patients is ongoing [NCT04114981] with results expected by the end of 2022.

### Radiotherapy techniques

SRS for < 10 mm BM is a high-precision treatment that requires a high level of technology. SRS can be delivered using different machines, with invasive contention or frameless, photons X or gamma. Several decades ago, in 1968, the Gamma Knife (GK) was introduced as the new treatment modality for SRS. The GK is a frame-based SRS that uses 60Co sources for irradiating a tumor volume with a diameter of approximately 4, 8, or 14 mm [75]. GK is mainly characterized by non-homogeneous dose distributions within the target due to the effect of overlapping shots. The Cyberknife (CK) was invented at Stanford Health Care and first debuted in 1994. CK is an image-guided frameless robotic technology designed to deliver non-isocenter non-coplanar beam, and the entire treatment procedure is completely non-invasive [76]. Despite the differences in treatment planning and dose delivery significant differences were not found in the quality of clinical outcome between GK and CK after SRS [77, 78].

Linear accelerator (LINAC)-based radiosurgery was developed as an alternative to GK SRS, using a standard LINAC modified for stereotactic purposes. Recent technical advances have made LINAC-based SRS (using multiple non-coplanar intersecting arc) a patient friendly technique, non-invasive, allowing for accurate patient positioning and a short treatment time [79, 80]. Following the technical improvements in treatment planning systems, LINAC-based SRS was marketed as having acceptably similar precision, accuracy, and mechanical stability for the treatment of numerous and small BM. Accordingly, LINAC-based SRS has been rapidly disseminating in the community in the last decades [81] and despite the lack of systematic comparisons with GK-SRS, clinical results appear to be similar [82].

LINAC-based SRS is considered a changing practice pattern in the treatment of BM NSCLC [83], considering also the benefit in the cost-effectiveness analysis compared to GK o CK SRS.

### Treatment strategies for critical areas

Additional comments are needed for BM in critical CNS areas including the brainstem and optic pathway. Brainstem lesions are rare (3–5% of all BM [84]) and surgery is not amenable for high-risk mortality or further functional impairment. Brainstem metastases come with a poor prognosis and estimated survival without treatment is dramatically poor (from one to six months) [85]. SRS is recommended for the treatment of brainstem metastases with a median dosage of 16 Gy (range 11–39) and median fractions 1 (1–13) [86]. In a recent large metanalysis including 15,900 brainstem metastases treated with SRS the 1-year LC was 86% with an objective response rate of 59% and symptoms improvement of 55%. The grade 3–5 toxicity was 2.4% and deaths from progression after SRS are rare [86].

Isolated optic nerve metastases are similarly rare but result in a unilateral or bilateral loss of the visual field. Thanks to the experience on gliomas and perioptic tumors [90, 91] prompt fractionation or multi-session radiosurgery is an option for treating this subset of patients with the risk of 1–2% of visual complication [87]. These favorable results suggest the feasibility of a local treatment in patients with NSCLC critical areas metastases regardless molecular status and systemic therapy.

### Combined treatment strategies and choice of the optimal timing

The combination of RT and TKI for BMs is still controversial. Results of the perspective study by Jiang et al. showed no advantage of early WBRT to TKI over TKI alone [88]. The results of a recent retrospective study showed a trend to significant advantage (although no difference in OS) of RT and TKI combo vs. TKI alone in terms of median intracranial PFS (27.6 vs. 16.1 months; *p* = 0.053) [89]. A large meta-analysis including 1,041 unselected NSCLC with BMs from 9 retrospective studies and 1 randomized controlled trial and aimed to investigate the combination of WBRT with EGFR TKI vs. WBRT alone or EGFR TKI therapy alone showed the best hazard ratios for intracranial PFS in patients who received EGFR-TKI alone [95].

More recently, a retrospective analysis aimed to compare SRS + TKI vs. WBRT + TKI vs. TKI alone reported a significant advantage in term of iPFS and OS in the first group (23 vs. 24 vs. 17 months, respectively; *p* = 0.025) (46 vs. 30 vs. 25 months, respectively; *p* = 0.001) [90]. A retrospective cohort of patients harboring EGFR-activating mutation treated with consolidative local ablative treatment yielded improved OS after first-line TKI. Interestingly, the BM site significantly affected the improved survival achieved with additional local treatment vs patients receiving exclusive systemic treatment (38.2 versus 29.2 months, HR = 0.48, 95% CI 0.30–0.76, *p* = 0.002) [91].

Another meta-analysis provided the evidence that early RT in these patients offers a significant iPFS and OS advantage that is strictly correlated with the number of BMs, being the best results achieved in those with less than three brain lesions. On the contrary, no advantage was recorded in the other patients and those showing massive disease [92].

On these bases no conclusive therapeutic statements may be defined and early radiotherapy continues to have a fundament role in the treatment of NSCLC patients with BMs harboring EGFR activating mutations.

As for the possible prognostic advantage of an upfront RT treatment followed by TKI therapy, [90] out of a multicentric series of 351 patients with BM from EGFR mutated NSCLC, 100 patients were treated with SRS followed by TKI therapy achieved the best therapeutic results (median survival, respectively, 46, 30, and 25 months; *p* < 0.001), compared to 120 with WBRT followed by TKI, and 131 with TKI followed by SRS or WBRT at progression.

At multivariate analysis, prognostic features didn’t significantly differ between the upfront SRS and EGFR-TKI cohorts, whereas the WBRT cohort was more likely to have a less favorable prognosis (*p* = 0.001). Despite the risk of selection biases because SRS is usually adopted for a limited number of BMs, this study shows the safety and effectiveness of elective RT procedure within a multidisciplinary therapeutic approach and warrants further investigation.

The efficacy of concurrent radiotherapy and EGFR TKIs is still unclear. The results of a retrospective study involving 44 EGFR-mutant NSCLC who received concurrent radiotherapy and TKI [93], recorded frequent and severe AEs with two patients that had to discontinue the treatment due to grade ≥ 3 cutaneous toxicity [93]. Additionally, they also reported radiation-related AEs including included hydrocephalus (2 patients), pneumonitis (3 patients, one grade ≥ 3), myocarditis (1 patient), radiodermatitis (3 patients), laryngo-pharyngitis (2 patients), esophagitis (2 patients), and enteritis (1 patient) [93].

Preliminary reports suggested improved survival of NSCLC-patients bearing ALK-rearrangement and treated with radiotherapy for BM. The introduction of targeted treatment has improved the response of these patients although intrinsic radiosensitivity of ALK-rearranged cells seems to play a prevalent role [94]. Johung et al. suggested a median life expectancy of 49.5 months in BM patients receiving both ALK-targeted therapy and radiotherapy [95]. Adjunction of radiotherapy to first-generation ALK-TKI crizotinib significantly improved response rate and progression-free survival in patients with BMs in multiple studies [96, 97].

However, the therapeutic landscape is rapidly changing following the development of new generations of ALK-TKIs with enhanced capability to diffuse thorough the BBB. Although a benefit of radiotherapy in association with 2nd generation drugs as ceritinib or alectinib or 3rd generation drugs as lorlatinib (as upfront therapy or following progression after crizotinib) has not been shown, it should be pointed out that because of the small study populations and heterogeneous treatments with SRS and/or WBRT, these studies were not conclusive [98] and did not underpin the deferral of local treatment.

Radiotherapy and lorlatinib may act cooperatively by targeting different intracranial compartments [99, 100], and case reports suggest that lorlatinib might be effective in intracranial sites that are traditionally considered unfit for radiotherapy such as symptomatic leptomeningeal dissemination, leading to impressive disease response (“Lazarus Effect”) [101].

## Follow-up of the patients

Three-six months after radiotherapy and/or systemic therapy BMs were crucially followed up with MRI and assessed by applying the response evaluation criteria in solid tumors (RECIST) [102]. For naïve patients, according to ASCO guidelines for stage I-III NSCLC, brain MRI for routine surveillance should not be used in patients who have undergone curative-intent treatment [103]. Indeed, for patients with clinical stage III-IV disease, surveillance brain MRI performed 12 months after initial evaluation may be warranted [4]. The same recommendation is extended to EGFR mutation-positive NSCLC that had a higher incidence of BMs than in those with *EGFR* mutation-negative adenocarcinoma [4]. A retrospective study on BMs after SRS showed that lesion less than 100 mm^3^ in volume or 6 mm in diameter reaches a 100% LC, thus routine surveillance with brain imaging to diagnose new out of field lesions should be considered as part of the standard care in all stage lung cancer [104]. The Ontario Cancer Registry demonstrated that patients with NSCLC and higher socioeconomic status showed an improved 5-year OS because underwent greater MRI, lung resection, adjuvant or intravenous chemotherapy and palliative radiotherapy [105]. On the other hand, Vernon et al. created a model of comprehensive clinical staging in resectable lung cancer and evaluated the role of brain MRI: additional staging information were found in only four of 274 cases (1.5%). The results of comprehensive clinical staging with and without MRI were identical 98.8% of the cases and if brain MRI were removed from the staging algorithm, the total cost of staging in this population would have been 31.9% cheaper [106].

## Conclusions and take-home message

**Fig. 1:**
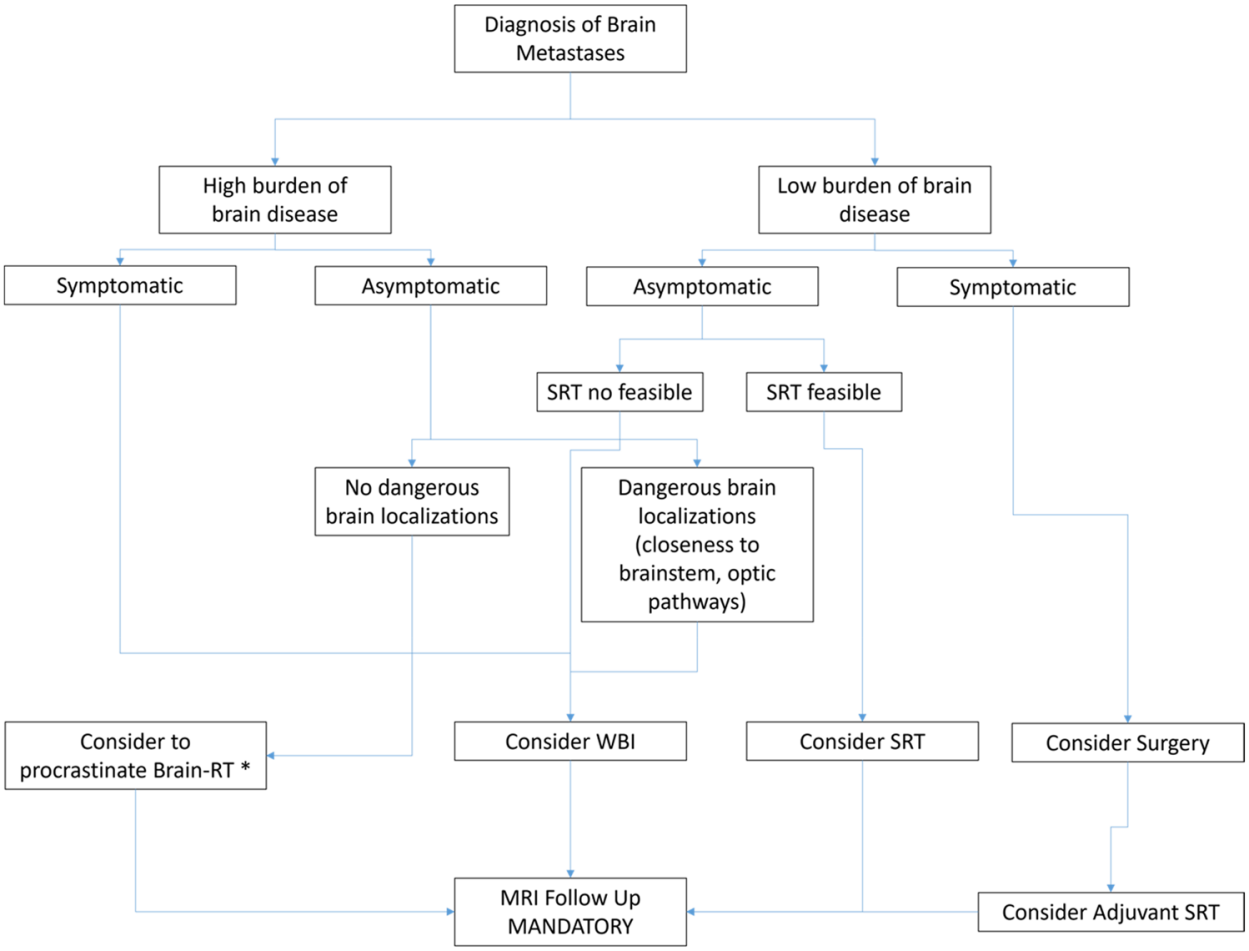
proposed workflow of clinical management of brain metastases in mNSCLC with druggable drivers’ mutations. *SRT* stereotactic radiotherapy, *WBI* whole brain irradiation, *MRI* magnetic resonance imaging; Systemic therapy with TKI should be considered for all the patients with brain metastases. *In this case it is mandatory the use of highly effect new generation TKI

In the light of what reviewed here, the treatment of BMs in patients with mNSCLC with or without druggable drivers’ mutations requires a personalized workflow and the presence of multiple professionals with proven experience. Figure 1 summarizes the proposed workflow of clinical management of BMs in patients with druggable mutation-driven mNSCLC. The current tumultuous development in this field disallows reaching guidelines set in stone. The large amounts of scientific information and the definition of specific clinical objectives musts be discussed case by case by a multidisciplinary team including the pathologist, neurosurgeon, neurologist, radiotherapist, oncologist and palliative care taking in full consideration that in the majority of cases the quality of life must the main target of the treatment strategy.

Not with standing, in the modern era of precision medicine, the opinion of all the authors is that brain MRI is fundamental: (a) for clinical staging in advanced or systemic metastatic NCSCL (b) for all patients with EGFR driver mutation that had a higher risk of developing BMs; (c) to estimate the intracranial progression to assess the need of tempestive treatment for new BMs after the local treatment (surgery and/or radiotherapy); (d) to follow up and control all the patients with BMs and driver mutation in which is considered safe and feasible to procrastinate a local treatment (i.e. surgery and/or RT).

## References

[1] Nicholson AG, Tsao MS, Beasley MB, Borczuk AC, Brambilla E, Cooper WA, Dacic S, Jain D, Kerr KM, Lantuejoul S (2022). The 2021 WHO classification of lung tumors: impact of advances since 2015. J Thorac Oncol.

[2] Siegel RL, Miller KD, Fuchs HE, Jemal A (2021). Cancer statistics, 2021. CA Cancer J Clin.

[3] Sung H, Ferlay J, Siegel RL, Laversanne M, Soerjomataram I, Jemal A, Bray F (2021). Global cancer statistics 2020: GLOBOCAN estimates of incidence and mortality worldwide for 36 cancers in 185 countries. CA Cancer J Clin.

[4] Kim M, Suh CH, Lee SM, Park JE, Kim HC, Kim SO, Aizer AA, Yanagihara TK, Bai HX, Guenette JP (2021). Development of brain metastases in patients with non-small cell lung cancer and no brain metastases at initial staging evaluation: cumulative incidence and risk factor analysis. AJR Am J Roentgenol.

[5] London D, Patel DN, Donahue B, Navarro RE, Gurewitz J, Silverman JS, Sulman E, Bernstein K, Palermo A, Golfinos JG (2021). The incidence and predictors of new brain metastases in patients with non-small cell lung cancer following discontinuation of systemic therapy. J Neurosurg.

[6] Nardone V, Giannicola R, Bianco G, Giannarelli D, Tini P, Pastina P, Falzea AC, Macheda S, Caraglia M, Luce A (2021). Inflammatory markers and procalcitonin predict the outcome of metastatic non-small-cell-lung-cancer patients receiving PD-1/PD-L1 immune-checkpoint blockade. Front Oncol.

[7] Pastina P, Nardone V, Croci S, Battaglia G, Vanni F, Bellan C, Barbarino M, Ricci V, Costantini S, Capone F et al. (2017) Anti-cancer activity of dose-fractioned mPE +/- bevacizumab regimen is paralleled by immune-modulation in advanced squamous NSLC patients. J Thorac Dis 10.21037/jtd.2017.08.68 10.21037/jtd.2017.08.68 PMC5708460 29221287

[8] Pastina P, Nardone V, Botta C, Croci S, Tini P, Battaglia G, Ricci V, Cusi MG, Gandolfo C, Misso G (2017). Radiotherapy prolongs the survival of advanced non-smallcell lung cancer patients undergone to an immune-modulating treatment with dose-fractioned cisplatin and metronomic etoposide and bevacizumab (mPEBev). Oncotarget.

[9] Martino EC, Misso G, Pastina P, Costantini S, Vanni F, Gandolfo C, Botta C, Capone F, Lombardi A, Pirtoli L (2016). Immune-modulating effects of bevacizumab in metastatic non-small-cell lung cancer patients. Cell Death Discov.

[10] Nadler E, Vasudevan A, Wang Y, Ogale S (2022). Real-world patterns of biomarker testing and targeted therapy in de novo metastatic non-small cell lung cancer patients in the US oncology network. Cancer Treat Res Commun.

[11] Wu Y, Verma V, Liang F, Lin Q, Zhou Z, Wang Z, Wang Y, Wang J, Chang JY (2022). Local consolidative therapy versus systemic therapy alone for metastatic non-small cell lung cancer: a systematic review and meta-analysis. Int J Radiat Oncol Biol Phys.

[12] Tan CS, Gilligan D, Pacey S (2015). Treatment approaches for EGFR-inhibitor-resistant patients with non-small-cell lung cancer. Lancet Oncol.

[13] Vogelbaum MA, Brown PD, Messersmith H, Brastianos PK, Burri S, Cahill D, Dunn IF, Gaspar LE, Gatson NTN, Gondi V (2022). Treatment for brain metastases: ASCO-SNO-ASTRO guideline. J Clin Oncol.

[14] Rangachari D, Yamaguchi N, VanderLaan PA, Folch E, Mahadevan A, Floyd SR, Uhlmann EJ, Wong ET, Dahlberg SE, Huberman MS (2015). Brain metastases in patients with EGFR-mutated or ALK-rearranged non-small-cell lung cancers. Lung Cancer.

[15] Hirsch FR, Scagliotti GV, Mulshine JL, Kwon R, Curran WJ, Wu YL, Paz-Ares L (2017). Lung cancer: current therapies and new targeted treatments. Lancet.

[16] Lynch TJ, Bell DW, Sordella R, Gurubhagavatula S, Okimoto RA, Brannigan BW, Harris PL, Haserlat SM, Supko JG, Haluska FG (2004). Activating mutations in the epidermal growth factor receptor underlying responsiveness of non-small-cell lung cancer to gefitinib. N Engl J Med.

[17] Liu X, Wang P, Zhang C, Ma Z (2017). Epidermal growth factor receptor (EGFR): a rising star in the era of precision medicine of lung cancer. Oncotarget.

[18] Ge M, Zhuang Y, Zhou X, Huang R, Liang X, Zhan Q (2017). High probability and frequency of EGFR mutations in non-small cell lung cancer with brain metastases. J Neurooncol.

[19] Heon S, Yeap BY, Britt GJ, Costa DB, Rabin MS, Jackman DM, Johnson BE (2010). Development of central nervous system metastases in patients with advanced non-small cell lung cancer and somatic EGFR mutations treated with gefitinib or erlotinib. Clin Cancer Res.

[20] Sekine A, Kato T, Hagiwara E, Shinohara T, Komagata T, Iwasawa T, Satoh H, Tamura K, Kasamatsu T, Hayashihara K (2012). Metastatic brain tumors from non-small cell lung cancer with EGFR mutations: distinguishing influence of exon 19 deletion on radiographic features. Lung Cancer.

[21] Shaw AT, Yeap BY, Mino-Kenudson M, Digumarthy SR, Costa DB, Heist RS, Solomon B, Stubbs H, Admane S, McDermott U (2009). Clinical features and outcome of patients with non-small-cell lung cancer who harbor EML4-ALK. J Clin Oncol.

[22] Solomon BJ, Mok T, Kim D-W, Wu Y-L, Nakagawa K, Mekhail T, Felip E, Cappuzzo F, Paolini J, Usari T (2014). First-line crizotinib versus chemotherapy in ALK-positive lung cancer. N Engl J Med.

[23] (2021) Lorlatinib Outperforms Crizotinib in NSCLC. Cancer Discov. 11(1):OF5. 10.1158/2159-8290.CDNB2020-110 10.1158/2159-8290.CD-NB2020-110 33298467

[24] Camidge DR, Dziadziuszko R, Peters S, Mok T, Noe J, Nowicka M, Gadgeel SM, Cheema P, Pavlakis N, de Marinis F (2019). Updated efficacy and safety data and impact of the EML4-ALK fusion variant on the efficacy of alectinib in untreated ALK-positive advanced non-small cell lung cancer in the global phase III ALEX study. J Thorac Oncol.

[25] Nishino M, Hatabu H (2017). Osimertinib in EGFR T790M-positive lung cancer. N Engl J Med.

[26] Ramalingam SS, Vansteenkiste J, Planchard D, Cho BC, Gray JE, Ohe Y, Zhou C, Reungwetwattana T, Cheng Y, Chewaskulyong B (2020). Overall survival with osimertinib in untreated, EGFR-mutated advanced NSCLC. N Engl J Med.

[27] Iuchi T, Shingyoji M, Sakaida T, Hatano K, Nagano O, Itakura M, Kageyama H, Yokoi S, Hasegawa Y, Kawasaki K (2013). Phase II trial of gefitinib alone without radiation therapy for Japanese patients with brain metastases from EGFR-mutant lung adenocarcinoma. Lung Cancer.

[28] Park SJ, Kim HT, Lee DH, Kim KP, Kim SW, Suh C, Lee JS (2012). Efficacy of epidermal growth factor receptor tyrosine kinase inhibitors for brain metastasis in non-small cell lung cancer patients harboring either exon 19 or 21 mutation. Lung Cancer.

[29] Wu YL, Zhou C, Cheng Y, Lu S, Chen GY, Huang C, Huang YS, Yan HH, Ren S, Liu Y (2013). Erlotinib as second-line treatment in patients with advanced non-small-cell lung cancer and asymptomatic brain metastases: a phase II study (CTONG-0803). Ann Oncol.

[30] Yang JC, Sequist LV, Geater SL, Tsai CM, Mok TS, Schuler M, Yamamoto N, Yu CJ, Ou SH, Zhou C (2015). Clinical activity of afatinib in patients with advanced non-small-cell lung cancer harbouring uncommon EGFR mutations: a combined post-hoc analysis of LUX-Lung 2, LUX-Lung 3, and LUX-Lung 6. Lancet Oncol.

[31] Yang JC, Sequist LV, Zhou C, Schuler M, Geater SL, Mok T, Hu CP, Yamamoto N, Feng J, O'Byrne K (2016). Effect of dose adjustment on the safety and efficacy of afatinib for EGFR mutation-positive lung adenocarcinoma: post hoc analyses of the randomized LUX-Lung 3 and 6 trials. Ann Oncol.

[32] Wu YL, Sequist LV, Tan EH, Geater SL, Orlov S, Zhang L, Lee KH, Tsai CM, Kato T, Barrios CH (2018). Afatinib as first-line treatment of older patients with EGFR mutation-positive non-small-cell lung cancer: subgroup analyses of the LUX-Lung 3, LUX-Lung 6, and LUX-Lung 7 trials. Clin Lung Cancer.

[33] Fassunke J, Müller F, Keul M, Michels S, Dammert MA, Schmitt A, Plenker D, Lategahn J, Heydt C, Brägelmann J (2018). Overcoming EGFR(G724S)-mediated osimertinib resistance through unique binding characteristics of second-generation EGFR inhibitors. Nat Commun.

[34] Hirashima T, Satouchi M, Hida T, Nishio M, Kato T, Sakai H, Imamura F, Kiura K, Okamoto I, Kasahara K (2019). Osimertinib for Japanese patients with T790M-positive advanced non-small-cell lung cancer: a pooled subgroup analysis. Cancer Sci.

[35] Carlisle JW, Ramalingam SS (2019). Improving outcomes for brain metastases in EGFR mutated NSCLC. Transl Lung Cancer Res.

[36] Ohe Y, Imamura F, Nogami N, Okamoto I, Kurata T, Kato T, Sugawara S, Ramalingam SS, Uchida H, Hodge R (2019). Osimertinib versus standard-of-care EGFR-TKI as first-line treatment for EGFRm advanced NSCLC: FLAURA Japanese subset. Jpn J Clin Oncol.

[37] Odogwu L, Mathieu L, Goldberg KB, Blumenthal GM, Larkins E, Fiero MH, Rodriguez L, Bijwaard K, Lee EY, Philip R (2018). FDA benefit-risk assessment of osimertinib for the treatment of metastatic non-small cell lung cancer harboring epidermal growth factor receptor T790M mutation. Oncologist.

[38] Planchard D, Brown KH, Kim DW, Kim SW, Ohe Y, Felip E, Leese P, Cantarini M, Vishwanathan K, Jänne PA (2016). Osimertinib Western and Asian clinical pharmacokinetics in patients and healthy volunteers: implications for formulation, dose, and dosing frequency in pivotal clinical studies. Cancer Chemother Pharmacol.

[39] Gray JE, Okamoto I, Sriuranpong V, Vansteenkiste J, Imamura F, Lee JS, Pang YK, Cobo M, Kasahara K, Cheng Y (2019). Tissue and plasma EGFR mutation analysis in the FLAURA trial: osimertinib versus comparator EGFR tyrosine kinase inhibitor as first-line treatment in patients with EGFR-mutated advanced non-small cell lung cancer. Clin Cancer Res.

[40] Ahn MJ, Han JY, Kim DW, Cho BC, Kang JH, Kim SW, Yang JC, Mitsudomi T, Lee JS (2020). Osimertinib in patients with T790M-positive advanced non-small cell lung cancer: Korean subgroup analysis from phase II studies. Cancer Res Treat.

[41] Sakai H, Hayashi H, Iwasa T, Hasegawa Y, Takeda M, Nakagawa K (2017). Successful osimertinib treatment for leptomeningeal carcinomatosis from lung adenocarcinoma with the T790M mutation of EGFR. ESMO Open.

[42] Lemjabbar-Alaoui H, Hassan OU, Yang YW, Buchanan P (2015). Lung cancer: biology and treatment options. Biochim Biophys Acta.

[43] Yamaguchi H, Wakuda K, Fukuda M, Kenmotsu H, Mukae H, Ito K, Chibana K, Inoue K, Miura S, Tanaka K (2021). A phase II study of osimertinib for radiotherapy-naive central nervous system metastasis from NSCLC: results for the T790M cohort of the OCEAN study (LOGIK1603/WJOG9116L). J Thorac Oncol.

[44] Yang Z, Guo Q, Wang Y, Chen K, Zhang L, Cheng Z, Xu Y, Yin X, Bai Y, Rabbie S (2016). AZD3759, a BBB-penetrating EGFR inhibitor for the treatment of EGFR mutant NSCLC with CNS metastases. Sci Transl Med.

[45] Noé J, Lovejoy A, Ou SI, Yaung SJ, Bordogna W, Klass DM, Cummings CA, Shaw AT (2020). ALK mutation status before and after alectinib treatment in locally advanced or metastatic ALK-positive NSCLC: pooled analysis of two prospective trials. J Thorac Oncol.

[46] Solomon BJ, Kim D-W, Wu Y-L, Nakagawa K, Mekhail T, Felip E, Cappuzzo F, Paolini J, Usari T, Tang Y (2018). Final overall survival analysis from a study comparing first-line crizotinib versus chemotherapy in ALK-mutation-positive non–small-cell lung cancer. J Clin Oncol.

[47] Blackhall F, Cappuzzo F (2016). Crizotinib: from discovery to accelerated development to front-line treatment. Ann Oncol.

[48] Soria JC, Tan DSW, Chiari R, Wu YL, Paz-Ares L, Wolf J, Geater SL, Orlov S, Cortinovis D, Yu CJ (2017). First-line ceritinib versus platinum-based chemotherapy in advanced ALK-rearranged non-small-cell lung cancer (ASCEND-4): a randomised, open-label, phase 3 study. Lancet.

[49] Okada K, Araki M, Sakashita T, Ma B, Kanada R, Yanagitani N, Horiike A, Koike S, Oh-Hara T, Watanabe K (2019). Prediction of ALK mutations mediating ALK-TKIs resistance and drug re-purposing to overcome the resistance. EBioMedicine.

[50] Barrows SM, Wright K, Copley-Merriman C, Kaye JA, Chioda M, Wiltshire R, Torgersen KM, Masters ET (2019). Systematic review of sequencing of ALK inhibitors in ALK-positive non-small-cell lung cancer. Lung Cancer (Auckl).

[51] Kim DW, Mehra R, Tan DSW, Felip E, Chow LQM, Camidge DR, Vansteenkiste J, Sharma S, De Pas T, Riely GJ (2016). Activity and safety of ceritinib in patients with ALK-rearranged non-small-cell lung cancer (ASCEND-1): updated results from the multicentre, open-label, phase 1 trial. Lancet Oncol.

[52] Felip E, Crinò L, Kim DW, Spigel DR, Nishio M, Mok T, Scagliotti G, Cesic D, Sutradhar S, Shaw AT (2016). 141PD: whole body and intracranial efficacy of ceritinib in patients (pts) with crizotinib (CRZ) pretreated, ALK-rearranged (ALK+) non-small cell lung cancer (NSCLC) and baseline brain metastases (BM): results from ASCEND-1 and ASCEND-2 trials. J Thorac Oncol.

[53] Naito T, Shiraishi H, Fujiwara Y (2021). Brigatinib and lorlatinib: their effect on ALK inhibitors in NSCLC focusing on resistant mutations and central nervous system metastases. Jpn J Clin Oncol.

[54] Garber K (2010). ALK, lung cancer, and personalized therapy: portent of the future?. J Natl Cancer Inst.

[55] Andratschke N, Kraft J, Nieder C, Tay R, Califano R, Soffietti R, Guckenberger M (2019). Optimal management of brain metastases in oncogenic-driven non-small cell lung cancer (NSCLC). Lung Cancer.

[56] Brown PD, Jaeckle K, Ballman KV, Farace E, Cerhan JH, Anderson SK, Carrero XW, Barker FG, Deming R, Burri SH (2016). Effect of radiosurgery alone vs radiosurgery with whole brain radiation therapy on cognitive function in patients with 1 to 3 brain metastases: a randomized clinical trial. Jama.

[57] Gaspar LE, Prabhu RS, Hdeib A, McCracken DJ, Lasker GF, McDermott MW, Kalkanis SN, Olson JJ (2019). Congress of neurological surgeons systematic review and evidence-based guidelines on the role of whole brain radiation therapy in adults with newly diagnosed metastatic brain tumors. Neurosurgery.

[58] Singh SA, McDermott DM, Mattes MD (2020). Impact of systemic therapy type and timing on intracranial tumor control in patients with brain metastasis from non-small-cell lung cancer treated with stereotactic radiosurgery. World Neurosurg.

[59] Minniti G, Le Rhun E (2022). Should radiotherapy be considered for the initial treatment of brain metastases?. Lancet Oncol.

[60] Mahajan A, Ahmed S, McAleer MF, Weinberg JS, Li J, Brown P, Settle S, Prabhu SS, Lang FF, Levine N (2017). Post-operative stereotactic radiosurgery versus observation for completely resected brain metastases: a single-centre, randomised, controlled, phase 3 trial. Lancet Oncol.

[61] Patchell RA, Tibbs PA, Regine WF, Dempsey RJ, Mohiuddin M, Kryscio RJ, Markesbery WR, Foon KA, Young B (1998). Postoperative radiotherapy in the treatment of single metastases to the brain: a randomized trial. JAMA.

[62] Brown PD, Ballman KV, Cerhan JH, Anderson SK, Carrero XW, Whitton AC, Greenspoon J, Parney IF, Laack NNI, Ashman JB (2017). Postoperative stereotactic radiosurgery compared with whole brain radiotherapy for resected metastatic brain disease (NCCTG N107C/CEC·3): a multicentre, randomised, controlled, phase 3 trial. Lancet Oncol.

[63] Ernani V, Stinchcombe TE (2019). Management of brain metastases in non-small-cell lung cancer. J Oncol Pract.

[64] Kim KM, Lee SH, Kim SM, Kim NY, Gwak HS, Shin SH, Kwon JW, Yoo H (2019). Discordance of epidermal growth factor receptor mutation between brain metastasis and primary non-small cell lung cancer. Brain Tumor Res Treat.

[65] Han C, Zou H, Ma J, Zhou Y, Zhao J (2010). Comparison of EGFR and KRAS status between primary non-small cell lung cancer and corresponding metastases: a systematic review and meta-analysis. Zhongguo Fei Ai Za Zhi.

[66] Yamamoto M, Serizawa T, Higuchi Y, Sato Y, Kawagishi J, Yamanaka K, Shuto T, Akabane A, Jokura H, Yomo S (2017). A multi-institutional prospective observational study of stereotactic radiosurgery for patients with multiple brain metastases (JLGK0901 study update): irradiation-related complications and long-term maintenance of mini-mental state examination scores. Int J Radiat Oncol Biol Phys.

[67] Balasubramanian SK, Sharma M, Venur VA, Schmitt P, Kotecha R, Chao ST, Suh JH, Angelov L, Mohammadi AM, Vogelbaum MA (2020). Impact of EGFR mutation and ALK rearrangement on the outcomes of non-small cell lung cancer patients with brain metastasis. Neuro Oncol.

[68] Quigley MR, Bello N, Jho D, Fuhrer R, Karlovits S, Buchinsky FJ (2015) Estimating the additive benefit of surgical excision to stereotactic radiosurgery in the management of metastatic brain disease. Neurosurgery 76: 707–712; discussion 712–703. 10.1227/neu.0000000000000707 10.1227/NEU.0000000000000707 25734321

[69] Prabhu RS, Press RH, Patel KR, Boselli DM, Symanowski JT, Lankford SP, McCammon RJ, Moeller BJ, Heinzerling JH, Fasola CE (2017). Single-fraction stereotactic radiosurgery (SRS) alone versus surgical resection and SRS for large brain metastases: a multi-institutional analysis. Int J Radiat Oncol Biol Phys.

[70] Jhaveri J, Chowdhary M, Zhang X, Press RH, Switchenko JM, Ferris MJ, Morgan TM, Roper J, Dhabaan A, Elder E (2018). Does size matter? investigating the optimal planning target volume margin for postoperative stereotactic radiosurgery to resected brain metastases. J Neurosurg.

[71] Choi CY, Chang SD, Gibbs IC, Adler JR, Harsh GRT, Lieberson RE, Soltys SG (2012). Stereotactic radiosurgery of the postoperative resection cavity for brain metastases: prospective evaluation of target margin on tumor control. Int J Radiat Oncol Biol Phys.

[72] Blonigen BJ, Steinmetz RD, Levin L, Lamba MA, Warnick RE, Breneman JC (2010). Irradiated volume as a predictor of brain radionecrosis after linear accelerator stereotactic radiosurgery. Int J Radiat Oncol Biol Phys.

[73] Minniti G, Clarke E, Lanzetta G, Osti MF, Trasimeni G, Bozzao A, Romano A, Enrici RM (2011). Stereotactic radiosurgery for brain metastases: analysis of outcome and risk of brain radionecrosis. Radiat Oncol.

[74] Milano MT, Grimm J, Niemierko A, Soltys SG, Moiseenko V, Redmond KJ, Yorke E, Sahgal A, Xue J, Mahadevan A (2021). Single- and multifraction stereotactic radiosurgery dose/volume tolerances of the brain. Int J Radiat Oncol Biol Phys.

[75] Wu A, Lindner G, Maitz AH, Kalend AM, Lunsford LD, Flickinger JC, Bloomer WD (1990). Physics of gamma knife approach on convergent beams in stereotactic radiosurgery. Int J Radiat Oncol Biol Phys.

[76] Wowra B, Muacevic A, Tonn JC (2012). CyberKnife radiosurgery for brain metastases. Prog Neurol Surg.

[77] Wowra B, Muacevic A, Tonn JC (2009). Quality of radiosurgery for single brain metastases with respect to treatment technology: a matched-pair analysis. J Neurooncol.

[78] Fareed MM, Eldib A, Weiss SE, Hayes SB, Li J, Ma CC (2018). A treatment planning comparison between a novel rotating gamma system and robotic linear accelerator based intracranial stereotactic radiosurgery/radiotherapy. Phys Med Biol.

[79] Deinsberger R, Tidstrand J (2005) Linac radiosurgery as a tool in neurosurgery. Neurosurg Rev 28: 79–88; discussion 89–90, 91. 10.1007/s10143-005-0376-7 10.1007/s10143-005-0376-7 15726439

[80] Niranjan A, Maitz AH, Lunsford A, Gerszten PC, Flickinger JC, Kondziolka D, Lunsford LD (2007). Radiosurgery techniques and current devices. Prog Neurol Surg.

[81] Harada K, Igaki H, Abe E, Ariga T, Hayashi N, Kanemoto A, Komiyama T, Matsumoto Y, Nakano T, Onimaru R (2018). Present clinical practices of stereotactic irradiation for metastatic brain tumors in Japan: results of questionnaire survey of the Japanese radiation oncology study group (JROSG) working subgroup for neurological tumors. Int J Clin Oncol.

[82] Alongi F, Fiorentino A, Mancosu P, Navarria P, Giaj Levra N, Mazzola R, Scorsetti M (2016). Stereotactic radiosurgery for intracranial metastases: linac-based and gamma-dedicated unit approach. Expert Rev Anticancer Ther.

[83] Park HS, Wang EH, Rutter CE, Corso CD, Chiang VL, Yu JB (2016). Changing practice patterns of Gamma Knife versus linear accelerator-based stereotactic radiosurgery for brain metastases in the US. J Neurosurg.

[84] Yoo TW, Park ES, Kwon DH, Kim CJ (2011). Gamma knife radiosurgery for brainstem metastasis. J Korean Neurosurg Soc.

[85] Fuentes S, Delsanti C, Metellus P, Peragut JC, Grisoli F, Regis J (2006) Brainstem metastases: management using gamma knife radiosurgery. Neurosurgery 58:37–42; discussion 37–42. 10.1227/01.neu.0000190655.95669.5c 10.1227/01.neu.0000190655.95669.5c 16385327

[86] Chen WC, Baal UH, Baal JD, Pai JS, Boreta L, Braunstein SE, Raleigh DR (2021). Efficacy and safety of stereotactic radiosurgery for brainstem metastases: a systematic review and meta-analysis. JAMA Oncol.

[87] Noël G, Antoni D, Barillot I, Chauvet B (2016). Delineation of organs at risk and dose constraints. Cancer Radiother.

[88] Jiang T, Su C, Li X, Zhao C, Zhou F, Ren S, Zhou C, Zhang J (2016). EGFR TKIs plus WBRT demonstrated no survival benefit other than that of TKIs alone in patients with NSCLC and EGFR mutation and brain metastases. J Thorac Oncol.

[89] Wang C, Lu X, Zhou Z, Wang J, Hui Z, Liang J, Feng Q, Chen D, Xiao Z, Lv J (2019). The efficacy of upfront intracranial radiation with TKI compared to TKI alone in the NSCLC patients harboring EGFR mutation and brain metastases. J Cancer.

[90] Magnuson WJ, Lester-Coll NH, Wu AJ, Yang TJ, Lockney NA, Gerber NK, Beal K, Amini A, Patil T, Kavanagh BD (2017). Management of brain metastases in tyrosine kinase inhibitor-naïve epidermal growth factor receptor-mutant non-small-cell lung cancer: a retrospective multi-institutional analysis. J Clin Oncol.

[91] Xu Q, Zhou F, Liu H, Jiang T, Li X, Xu Y, Zhou C (2018). Consolidative local ablative therapy improves the survival of patients with synchronous oligometastatic NSCLC harboring EGFR activating mutation treated with first-line EGFR-TKIs. J Thorac Oncol.

[92] Wang C, Lu X, Lyu Z, Bi N, Wang L (2018). Comparison of up-front radiotherapy and TKI with TKI alone for NSCLC with brain metastases and EGFR mutation: a meta-analysis. Lung Cancer.

[93] Wang Y, Li Y, Xia L, Niu K, Chen X, Lu D, Kong R, Chen Z, Sun J (2018). Continued EGFR-TKI with concurrent radiotherapy to improve time to progression (TTP) in patients with locally progressive non-small cell lung cancer (NSCLC) after front-line EGFR-TKI treatment. Clin Transl Oncol.

[94] Mak KS, Gainor JF, Niemierko A, Oh KS, Willers H, Choi NC, Loeffler JS, Sequist LV, Shaw AT, Shih HA (2015). Significance of targeted therapy and genetic alterations in EGFR, ALK, or KRAS on survival in patients with non-small cell lung cancer treated with radiotherapy for brain metastases. Neuro Oncol.

[95] Johung KL, Yeh N, Desai NB, Williams TM, Lautenschlaeger T, Arvold ND, Ning MS, Attia A, Lovly CM, Goldberg S (2016). Extended survival and prognostic factors for patients with ALK-rearranged non-small-cell lung cancer and brain metastasis. J Clin Oncol.

[96] Costa DB, Shaw AT, Ou SH, Solomon BJ, Riely GJ, Ahn MJ, Zhou C, Shreeve SM, Selaru P, Polli A (2015). Clinical experience with crizotinib in patients with advanced ALK-rearranged non-small-cell lung cancer and brain metastases. J Clin Oncol.

[97] Yoshida T, Oya Y, Tanaka K, Shimizu J, Horio Y, Kuroda H, Sakao Y, Hida T, Yatabe Y (2016). Clinical impact of crizotinib on central nervous system progression in ALK-positive non-small lung cancer. Lung Cancer.

[98] Gadgeel SM, Shaw AT, Govindan R, Gandhi L, Socinski MA, Camidge DR, De Petris L, Kim DW, Chiappori A, Moro-Sibilot DL (2016). Pooled analysis of CNS response to alectinib in two studies of pretreated patients with ALK-positive non-small-cell lung cancer. J Clin Oncol.

[99] Wang W, Sun X, Hui Z (2019). Treatment optimization for brain metastasis from anaplastic lymphoma kinase rearrangement non-small-cell lung cancer. Oncol Res Treat.

[100] Ceddia S, Codacci-Pisanelli G (2021). Treatment of brain metastases in ALK-positive non-small cell lung cancer. Crit Rev Oncol Hematol.

[101] Facchinetti F, Levy A, Ammari S, Naltet C, Lavaud P, Aldea M, Vasseur D, Planchard D, Besse B (2021). Meningeal, "Lazarus Response" to lorlatinib in a ROS1-positive NSCLC patient progressing to entrectinib. Cancer Manag Res.

[102] Eisenhauer EA, Therasse P, Bogaerts J, Schwartz LH, Sargent D, Ford R, Dancey J, Arbuck S, Gwyther S, Mooney M (2009). New response evaluation criteria in solid tumours: revised RECIST guideline (version 1.1). Eur J Cancer.

[103] Schneider BJ, Ismaila N, Aerts J, Chiles C, Daly ME, Detterbeck FC, Hearn JWD, Katz SI, Leighl NB, Levy B (2020). Lung cancer surveillance after definitive curative-intent therapy: ASCO guideline. J Clin Oncol.

[104] Wolf A, Kvint S, Chachoua A, Pavlick A, Wilson M, Donahue B, Golfinos JG, Silverman J, Kondziolka D (2018). Toward the complete control of brain metastases using surveillance screening and stereotactic radiosurgery. J Neurosurg.

[105] Shah M, Parmar A, Chan KKW (2020). Socioeconomic disparity trends in diagnostic imaging, treatments, and survival for non-small cell lung cancer 2007–2016. Cancer Med.

[106] Vernon J, Andruszkiewicz N, Schneider L, Schieman C, Finley CJ, Shargall Y, Fahim C, Farrokhyar F, Hanna WC (2016). Comprehensive clinical staging for resectable lung cancer: clinicopathological correlations and the role of brain MRI. J Thorac Oncol.

